# Mobile money transactions and banking sector performance in Ghana

**DOI:** 10.1016/j.heliyon.2022.e10761

**Published:** 2022-09-29

**Authors:** Evans Kulu, Alexander Opoku, Emmanuel Gbolonyo, Mark Anthony Tayi Kodwo

**Affiliations:** a C.K. Tedam University of Technology and Applied Sciences, Department of Industrial Mathematics, Navrongo, Ghana; b University of Cape Coast, Department of Applied Economics, School of Economics, Cape Coast, Ghana; c University of Cape Town, School of Economics, Cape Town, South Africa; d Bank of Ghana, Fin Tech and Innovation Office, Accra, Ghana

**Keywords:** ARDL, Bank sector performance, ECM, Ghana, Mobile money transaction

## Abstract

This study examines the effect of mobile money transactions on banking sector performance in Ghana using monthly data for the period 2015–2020. A composite index of banking sector performance was created from three indicators namely; depth, stability and efficiency of the banking sector. Using the Autoregressive Distributed Lag (ARDL) bounds test for cointegration, the Error Correction Model (ECM) and impulse responses analysis, the findings revealed that mobile money transaction has a detrimental effect on banking sector performance. Findings from the disaggregated components indicate that mobile money transactions hurt banking sector efficiency, while its effect on banking sector depth and stability is insignificant in the long run. It is concluded that the implication varies from one indicator to another concerning the direction and magnitude of the influence. The study, therefore, recommends that banks exercise much caution in their decision to consider adopting mobile money-based services when designing their business models.

## 1 Introduction

The majority of the economic activities that are undertaken involve the use of money. Regardless of the location, there is the need for business partners dealing in the exchange of goods and services to rely on the use of money all other things being equal. This, therefore, makes the role of the banking sector very essential. Among other functions, the traditional banking system facilitates the transfer of money from one person to another through varied services available. Indeed, people’s decision on whether or not to do business with a particular bank is dependent on how reliable they are in rendering this service (Kulu and Appiah-Kubi, 2021). However, the cost of money transfer transactions that are made through bank and non-bank transfer systems over the years has been relatively expensive, particularly for small amounts for both international and domestic transactions (Kirui et al., 2013). Receiving or transferring money is a vital activity in the daily lives of the average Ghanaian. Money transfer also needs a reliable, effective and affordable mobile transfer service that sees to it that money deposited in one location can be withdrawn in another location (Opare, 2018).

A mobile money system is a financial tool that makes use of mobile phones in sending and or in receiving money from far and wide within real-time. To make this possible, both the receiver and the sender need to have a virtual account on their phone with the respective service providers. One typical feature that makes the service popular is the fact that providers of the service are relatively available on every corner of the street even in geographically remote areas (Gosavi, 2018). In Ghana, the service providers include MTN, Vodafone and AirtelTigo respectively in charge of MTN mobile money (momo), Vodafone cash and AirtelTigo cash. This system is introduced in Ghana to push the cashless economy agenda ongoing. Muisyo et al. (2014a) argue that the incidence of robbery and theft associated with the informal system of transferring money through courier companies in a form of letters and parcels are minimized. The mobile money service is also known to help reduce the challenges associated with the traditional banks in the form of delays at the banking halls, joining of long queues, failure in branch networks, bank insolvency, unreliable channels of communications, and the likes (Gosavi, 2018).

The banking system in Ghana keeps expanding with time as new banking products and innovations are being introduced. Also, mobile money service in Ghana is witnessing expansion in terms of the number of subscribers, and merchants owing to some new products introduced and their convenience. Given that the two systems almost provide the same service, is the mobile money service serving as a substitute for the banking service hence a fall in the latter’s performance? Indeed, the banking system is relevant and can be structured to perform even better amidst the success of the mobile money system. In the Ghanaian economy where the role of finance is vital, there is the need to investigate the relationship between these two systems. Thus, authorities are making significant efforts in safeguarding and improving the services of both mobile money and the banking sector. For instance, the mobile money interoperability and other links or services performed by the mobile money initiative and the banking sector are commendable (Aker and Wilson, 2013). However, there is a paucity of studies in the context of Ghana investigating the effect of mobile money transactions on the performance of the banking sector as an effort to provide empirical evidence on the effect and possible recommendations aimed at guiding them in the right direction. Our study is therefore geared toward filling this research gap, and thus, this focus is in the right direction in helping to achieve economic stability and improving the overall performance of the financial sector.

The rest of the study is organised as follows; the literature review is presented in the next section, followed by the data and methodology, the results and discussions and finally the conclusions and policy recommendations.

## 2 Literature review

### 2.1 Theory and evidence

The theoretical underpinning of the link between mobile money and bank performance is based on the theory on innovation (Schumpeter, 2008) and technology acceptance models; namely the technology acceptance model (TAM), the theory of planned behavior (TPB) and the theory of reasoned action (TRA) (see Yousafzai et al., 2010; Asongu and Odhiambo, 2020; Asongu et al., 2018; Asongu et al., 2021). Schumpeter proposed that innovation is the basis of profitability and competitive advantage. The three attendant theories – TPB, TAM, and TRA that underpin this study argue that the decision to choose, adapt, and employ a specific technology is influenced by the type of technology as well as the expected innovation externalities associated with the underlying technology. According to the TRA, users of technological innovations, such as the mobile money are not oblivious of their actions and have rational intentions in relation to the adoption of these technologies (Fishbein and Ajzen, 1975; Ajzen and Fishbein, 1980; Bagozzi, 1982).

The TPB, which is an extension of the TRA, explains that there are variations among people who appear to have some conscious relations to their actions versus those who do not (Asongu et al., 2021). Thus, individuals' choice to use a particular technology innovation (as mobile money in this study) is gauged for its benefits in relation to the behavior and population of interest. According to the TAM, the assumption that someone will want to adopt and use a particular technological innovation depends on that person consciously considering and choosing to do so because of its usefulness and convenience (Davis, 1989).

Contextually, consistent with the three theories discussed, mobile money as an innovation in many African countries outperform its money-transfer predecessors in almost every way. Indeed, users perceive that it is faster, cheaper, more dependable, and safer. Accordingly, given the long history of cash-based transactions in sub-Saharan Africa and the prevalence of cash as the primary form of payment, users of mobile money users are likely to cause them considerable harm if it were to be turned off. Moreover, in a setting like Africa where a significant number of people do not have a bank account or access to basic financial service (Wambari, 2009), it implies that the presence of mobile money has the potential of offering banking products through mobile phones to people who transact or do businesses with no bank accounts.

However, whether or not mobile money transactions improves or harms banking sector performance (i.e., complement or substitute traditional banking) remains uncertain. On the one hand, mobile money transactions improve banking sector performance because (1) mobile money and banks are considered to serve different purposes and are not substitutes, which means that commercial banks can continue to expand as mobile money upsurges (2) mobile money and commercial banking are complementary as mobile money helps to mobilize deposits and eventually enables customers to use more commercial bank services. This may also lower the unit cost of financial services because mobile money products are linked to official bank accounts, allowing banks to increase their customer base and product selection (Kipkemboi and Bahia, 2019). On the other hand, given the widespread acceptance of mobile money transactions, the majority of funds might stay outside the banking industry for a while. As a result, unbanked individuals will now use electronic wallets in place of traditional bank accounts. The number of credits in a bank’s assets decreases, which lowers the profits that can be made from the assets. Consequently, a decline in revenues has the potential to affect how well the banking industry performs.

A substantial body of research contends that mobile money is essential to understanding the performance of financial institutions, especially Small and Medium-sized enterprises (SMEs) and banks. However, there is not a lot of empirical evidence, particularly in the context of Ghana. By examining the impact of mobile money transactions on the performance of banks, this study contributes to the body of literature. Indeed, several studies have thus far not thoroughly examined the problems as done by this study. Moreover, the extant literature undertaken on the subject matter have focused mostly on qualitative description of the influence of mobile money or mobile services on either bank performance or SME performance. For instance, Muisyo et al., 2014b, Muisyo et al., 2014a examined the effects of mobile money services on the performance of banking institutions in Kakamega town, Kenya and concluded that provision of mobile money services by various service providers has a positive impact on the performance of the banking institutions. Similarly, Nyaga (2017) investigated the influence of mobile money services on SME performance and documented that mobile money usage has made a major beneficial contribution to the SME sector since it is preferred by the majority of traders over the official banking sector for daily transactions. A similar thought is shared by Nyathira (2012) who argues that mobile money, as a financial innovation, contribute favourably to profitability of commercial banks.

Kirui and Onyuma (2015) added to the body of knowledge by investigating the impact of mobile money transactions on SME sales turnover in Nakuru Town. Using a descriptive cross-sectional survey approach to target 21,139 registered SMEs in Kenya’s Nakuru Town Sub-Counties, the results from their regression unveiled that mobile money transactions has a positive significant relationship with SME sales turnover. In a related study, Ahmed and Wamugo (2018) found that financial innovations such as agency banking, mobile banking, internet banking, and ATM banking have a positive and significant impact on commercial banks' performance in Kenya via a variety of channels, including increased profitability, reduced banking and other infrastructure costs, increased productivity and efficiency, increased customer outreach and customer relationship management, and increased accessibility. A similar outcome was obtained by Harelimana (2017) who analysed the effect of mobile banking in financial performance of Unguka Bank Limited, Rwanda from the period spanning from 2012 to 2016. Likewise, Kisaka et al. (2015) investigated the link between mobile banking deepening and commercial bank financial performance in Kenya. The study used a descriptive research design to cover six communications service providers and 43 commercial banks operating in Kenya. The authors found that there was a positive relationship between total number of mobile banking users and the value of mobile banking transactions although the study found a weak negligible positive relationship between mobile banking deepening and commercial bank financial performance. This is in line with a study by Kithaka (2014). In Douala, Cameroon, Talom and Tengeh (2020) examined the impact of the mobile money payment and receipt services on financial performance. The researchers concluded that the adoption of Mobile Money services exerted a significantly positive effect on the financial performance (i.e., sales turnover) of SMEs.

On the other hand, Kamukama and Tumwine (2012) find that increased use of mobile money services is associated with lowered bank deposits by clients, which negatively affected commercial banks' liquidity position, using an Ordinary Least Square (OLS) cross sectional and quantitative research design on 345 respondents from 23 commercial banks in Uganda. The authors also report that commercial banks' liquidity positions were deteriorating, with mobile money services accounting for 36.7 percent of the variation in commercial bank liquidity. A similar conclusion was obtained by Iheanachor and Ozegbe (2020) who investigated the causal link between mobile money and bank performance in Nigeria by applying the Autoregressive Distributed Lag (ARDL) model and Wald causality for a quarterly time-series data from 2014 to 2018. The study revealed that despite a steady increased in the volume and value of mobile money transactions empirical evidence from the analyses showed that mobile money variable hinders rather than helps banks' profitability. The authors also show that mobile money does not make a major contribution to the financial institutions' current assets base.

Notwithstanding, an empirical investigating as to whether mobile money transaction has aided the performance of the Ghanaian banking sector is nonexistent in the literature. The argument follows from fact that Ghana’s financial sector is not effectively executing its development mandates especially following recent reforms by the Bank of Ghana to clean up defunct banks and as such and it is currently not in a position to fulfil its potential as a dependable driver of economic growth and development. The closest to the scholarship in the case of Ghana is the study by Opare (2018) who only provides an essay on merits and demerits of mobile money on the profitability of the Ghanaian banking industry without any empirical framework.

The contention of this paper is that the omitted variable bias, technique(s) adopted and, more specifically, the context under consideration, could be responsible for the inconsistent nature of many of the results reported by previous researchers. These concerns necessitate the present study. Thus, our study fills this gap by providing rigorous econometric analysis within scope of the study. To the best of our knowledge, this is the only study in the context of Ghana that examines the effects of mobile money transactions on banking sector performance within ARDL framework.

## 3 Data and methodology

### 3.1 Data

The study uses monthly data spanning the period March 2015–December 2020. The banking sector’s performance is measured by a composite index created from various indicators of banking sector development using the Principal Component Analysis (PCA) technique, which is widely used in the literature. PCA is a simple and effective method for reducing a dataset to lower dimensionality while maintaining as much information from the original set as possible. It accomplishes this by generating new uncorrelated variables that maximize variance sequentially. It also contributes to the reduction of multicollinearity in modeling. Three dimensions of banking sector development were considered for the index construction, thus, depth, efficiency, and stability, as developed by Cihak et al. (2013). The depth measure was proxied with private sector credit. The efficiency measure was proxied with return on asset. The stability measure was proxied with the capital adequacy ratio. In the original form of the measures of banking sector development, the first principal component was extracted as the composite index of banking sector development (see Appendix B for summary statistics of the first principal components). All the data used was obtained from the Bank of Ghana (BoG) monetary time series database. These are macroeconomics variables that the Central Bank of Ghana publish on the monetary and financial system. It is a comprehensive database of all the licensed Banks, Savings and Loans, Leasing Companies and other bank and financial institutions. Appendix A presents the definition and measurement of the variable.

### 3.2 Theoretical model specification

The intuition behind the relationship between mobile money transaction and banking sector performance stems from the hypothesis that, the adoption of technological innovation (mobile money) in line with the TAM, TRA and TPB theories, implies less banking activities within the banking sector. Specifically, the concern of bank accounts and attendant dynamics of not having the expected effect on financial inclusion in developing countries (Asongu et al., 2021), suggest that with the use of mobile money, there would be more cash in hand for individual and less funds for commercial banks. That is, a trade-off between mobile money transaction and banking sector performance can be modelled in the form;

(1) BSP=f(MOMO,V)

where *BSP* is the banking sector performance measured by a composite index, MOMO represents mobile money transactions, V denotes other determining variables.

### 3.3 Empirical model specification

Using country-level aggregate data, we estimate banking sector performance as a function of mobile money transactions and other covariates. To test the relationship between mobile money transaction and banking sector performance as well as other macroeconomic determinants, we expand V from Eq. (1) to capture other drivers of banking sector performance and obtain Eq. (2) as;

(2) $$BSP_{t}=\gamma_{0}+\gamma_{1}MOMO_{t}+\gamma_{2}IR_{t}+\gamma_{3}SIZE_{t}+\gamma_{4}INF_{t}+\gamma_{5}DDEBT_{t}+\gamma_{6}EA_{t}+\mu_{t}\;$$

where BSP is banking sector performance measured by a composite index, MOMO denotes mobile money transactions, IR denotes Interest Rate, SIZE denotes banking sector size, INF denotes inflation, DDEBT denotes domestic debt, EA denotes economic activity. The coefficients $\gamma_{0}$, $\gamma_{1}$*,* $\gamma_{2}$*,* $\gamma_{3}$*,* $\gamma_{4}$*,* $\gamma_{5}$*,* and $\gamma_{6}$ are the elasticities of their respective variables, $\gamma_{0}$ is the constant component, the subscript t denotes time and $\mu_{t}$ is the error term [N(0,σ^2^)]. Looking at the effect of mobile money transactions on the disaggregated components of banking sector performance (depth, efficiency and stability), we specify the econometric models 3, 4 and 5. Thus, private sector credit, capital adequacy ratio and return on asset are used as the dependent variables in Eqs. (3), (4), and (5) respectively as:

(3) $$PSC_{t}=\gamma_{0}+\gamma_{1}MOMO_{t}+\gamma_{2}IR_{t}+\gamma_{3}SIZE_{t}+\gamma_{4}INF_{t}+\gamma_{5}DDEBT_{t}+\gamma_{6}EA_{t}+\mu_{t}$$

(4) $$CAR_{t}=\sigma_{0}+\sigma_{1}MOMO_{t}+\sigma_{2}IR_{t}+\sigma_{3}SIZE_{t}+\sigma_{4}INF_{t}+\sigma_{5}DDEBT_{t}+\sigma_{6}EA_{t}+\mu_{t}$$

(5) $$ROA_{t}=\varphi_{0}+\varphi_{1}MOMO_{t}+\varphi_{2}IR_{t}+\varphi_{3}SIZE_{t}+\varphi_{4}INF_{t}+\varphi_{5}DDEBT_{t}+\varphi_{6}EA_{t}+\mu_{t}$$

where $PSC_{t}\;$ represents private sector credit (used as a proxy for depth), $CAR_{t}\;$ represents capital adequacy ratio (used as a proxy for measuring stability) and $ROA_{t}\;$ represents return on asset, (used as a proxy for efficiency). The Autoregressive Distributed Lag (ARDL) model is used to respectively ascertain the relationship between mobile money transactions and banking sector performance.

The ARDL technique was used to determine the short-run and long-run relationship simultaneously without the problem of non-stationarity (Alih et al., 2018). The bounds test is also performed to determine whether a long-run relationship exists. The ARDL model specifications of the functional relationship between named variables are modeled in Eq. (6) as;

(6) $$DBSP_{t}=\delta_{0}+\overset{k}{\underset{i=1}{\sum }}\delta_{1}DBSP_{t-1}+\overset{k}{\underset{i=0}{\sum }}\delta_{2}LMOMO_{t-1}+\overset{k}{\underset{i=0}{\sum }}\delta_{3}IR_{t-1}+\overset{k}{\underset{i=0}{\sum }}\delta_{4}SIZE_{t-1}+\overset{k}{\underset{i=0}{\sum }}\delta_{5}LINF_{t-1}+\overset{k}{\underset{i=0}{\sum }}\delta_{6}DDEBT_{t-1}+\overset{k}{\underset{i=0}{\sum }}\delta_{7}EA_{t-1}+\gamma_{1}DBSP_{t-1}+\gamma_{2}MOMO_{t-1}+\gamma_{3\;}IR_{t-1}+\gamma_{4}SIZE_{t-1}+\gamma_{5}INF_{t-1}+\gamma_{6}DDEBT_{t-1}+\gamma_{7}EA_{t-1}+u_{t}$$

where k = lag order.

The ARDL-bound testing procedure permits us to take into consideration I(0) and I(1) variables together. The null hypothesis of the non-existence of a long-run relationship which is denoted by $F_{BSP\;}\left(LMOMO,\;IR,SIZE,LINF,DDEBT,LCIE\right)$. These are tested against the alternative hypothesis of the existence of co-integration:

$$H_{0}=\gamma_{1}=\gamma_{2}=\gamma_{3}=\gamma_{4}=\gamma_{5}=\gamma_{6}=\gamma_{7}=0$$

Against:

$$H_{1}\neq \gamma_{1}\neq \gamma_{2}\neq \gamma_{3}\neq \gamma_{4}\neq \gamma_{5}\neq \gamma_{6}\neq \gamma_{7}=0$$

The calculated F-statistics derived from the Wald test are compared with Pesaran et al.’s (2001) critical values. If the calculated F-statistics falls below the lower-bound critical values, then we fail to reject the null hypothesis of the non-existence of a long-run relationship. Moreover, if the calculated F-statistic lies between the lower- and upper-bound critical values, then the result is inconclusive. On the other hand, if the calculated F-statistics is more than the upper-bound critical values, then we reject the null hypothesis “non-existence of a long-run relationship”. Once the existence of a long-run relationship between variables is valued, the next step is to select the optimal lag length by using standard criteria such as Schwarz Bayesian criterion (SBC) or Akaike Information (AIC). Only after the test can the long and short-run coefficients be predicted. The ARDL long-run form is exhibited in Eq. (7):

(7) $$\begin{array}{l} BSP_{t}=\delta_{0}+\overset{k}{\underset{i=1}{\sum }}\delta_{1}BSP_{t-1}+\overset{k}{\underset{i=0}{\sum }}\delta_{2}MOMO_{t-1}+\overset{k}{\underset{i=0}{\sum }}\delta_{3}IR_{t-1}+\overset{k}{\underset{i=0}{\sum }}\delta_{4}SIZE_{t-1}\\ \;+\overset{k}{\underset{i=0}{\sum }}\delta_{5}INF_{t-1}+\overset{k}{\underset{i=0}{\sum }}\delta_{6}DDEBT_{t-1}+\overset{k}{\underset{i=0}{\sum }}\delta_{7}EA_{t-1}+u_{t}\; \end{array}$$

The error-correction term which was used in the ARDL short-run model to depict the short-run dynamic is shown in Eq. (8)

(8) $$\begin{array}{l} DBSP_{t}=\delta_{0}+\overset{k}{\underset{i=1}{\sum }}\delta_{1}DBSP_{t-1}+\overset{k}{\underset{i=0}{\sum }}\delta_{2}DMOMO_{t-1}+\overset{k}{\underset{i=0}{\sum }}\delta_{3}DIR_{t-1}+\overset{k}{\underset{i=0}{\sum }}\delta_{4}DSIZE_{t-1}\\ \;+\overset{k}{\underset{i=0}{\sum }}\delta_{5}DINF_{t-1}+\overset{k}{\underset{i=0}{\sum }}\delta_{6}DDDEBT_{t-1}+\overset{k}{\underset{i=0}{\sum }}\delta_{7}DEA_{t-1}+\delta_{8}ECT_{t-1}\; \end{array}$$

where, ECT = lagged error-correction term. We will be testing the null hypothesis ($H_{0}$) of the “non-existence of the long-run relationship” against the alternative of “the existence of the long-run relationship”

$$H_{0}=\delta_{1}=\delta_{2}=\delta_{3}=\delta_{4}=\delta_{5}=\delta_{6}=\delta_{7}=0$$

$$H_{1}\neq \delta_{1}\neq \delta_{2}\neq \delta_{3}\neq \delta_{4}\neq \delta_{5}\neq \delta_{6}\neq \delta_{7}\neq 0$$

## 4 Results and discussion

Table 1 shows the descriptive statistics of the variables used. The statistics for the variables were performed at the natural level of the variables. For the variables used, there is no evidence of outliers in the datasets since the mean values of the variables are higher than their respective standard deviations or there is a limited difference between the minimum and maximum values. On average, the total amount of mobile money transactions performed within the period under study is 18,979.35 Ghana cedis. Again, among the three measures of banking sector performance, it is observed that private sector credit is negatively skewed while capital adequacy ratio and return on asset are positively skewed. Also, in Appendix C, there is evidence of minimal correlation among the independent variables hence the models do not suffer the problem of multicollinearity.

**Table 1:** Descriptive statistics.

	BSP	PSC	CAR	ROA	MOMO	IR	SIZE	INF	D_DEBT	EA
Mean	0.000	26264.840	18.669	4.275	18979.350	25.645	24.538	13.677	24.958	3.533
Median	-0.225	30007.620	18.375	4.240	16553.980	25.930	24.890	14.245	24.720	3.510
Maximum	2.208	43533.190	23.890	6.920	67732.320	29.220	28.170	19.200	38.230	13.950
Minimum	-2.639	131.050	14.750	1.610	1805.900	20.950	19.120	7.800	0.000	-10.470
Std. Dev.	1.137	12865.060	1.788	0.916	15693.660	2.551	1.756	3.300	5.166	4.016
Skewness	-0.066	-1.140	0.278	0.136	1.287	-0.215	-0.626	-0.047	-1.002	-0.813
Kurtosis	2.338	3.227	2.873	3.818	4.208	1.653	3.203	1.671	9.577	6.157
Observations	72	72	72	72	72	72	72	72	72	72

The unit root test was done using the ADF and PP unit root test procedures to ascertain the stationarity properties of the variables used. This is presented in Table 2 and it indicates that some variables are stationary at levels while others are stationary at first difference. This is irrespective of whether the test is done with a constant and trend or with a constant but with no trend. The unit root results presented also show that aside from BSP,DDEBT, EA and ROA that are stationary at levels, the remaining variables are stationary at first difference. Therefore, the ARDL becomes the ideal model for the analysis after determining the presence of both I(0) and I(1) integration in the unit root test among the variables. Before proceeding with the test of co-integration, we determine the order of the vector autoregression (VAR), that is, the number of lags to be used. The optimum lags are automatically selected based on the Hannan-Quinn (HQ), Schwarz information criteria (SIC), and Akaike information criterion (AIC) (see Table 3).

**Table 2:** Unit root test results.

	ADF				PP			
	LEVEL		FIRST DIFFERENCE		LEVEL		FIRST DIFFERENCE	
Variables	Trend	No trend	Trend	No trend	Trend	No trend	Trend	No trend
BSP	-4.637∗∗∗	-3.171∗∗	-11.717∗∗∗	-11.765∗∗∗	-4.561∗∗∗	-2.938∗∗	-22.119∗∗∗	-18.362∗∗∗
PSC	-1.879	-1.915	-8.209∗∗∗	-8.251∗∗∗	-1.985	-2.001	-8.209∗∗∗	-8.251∗∗∗
CAR	-4.236∗∗∗	-2.494	-9.211∗∗∗	-9.280∗∗∗	-4.159∗∗∗	-2.282	-14.634∗∗∗	-14.803∗∗∗
ROA	-2.984	-2.972∗∗	-9.471∗∗∗	-9.399∗∗∗	-5.027∗∗∗	-4.366∗∗∗	-24.964∗∗∗	-20.633∗∗∗
MOMO	-2.689	-1.936	-11.499∗∗∗	-11.324∗∗∗	-2.481	-1.512	-11.661∗∗∗	-11.334∗∗∗
IR	-2.029	0.442	-14.241∗∗∗	-14.219∗∗∗	-3.037	0.219	-14.357∗∗∗	-14.222∗∗∗
SIZE	-5.580∗∗∗	-1.525	-6.937∗∗∗	-9.119∗∗∗	-5.562∗∗∗	-2.495	-46.889∗∗∗	-25.855∗∗∗
INF	-2.628	-1.917	-5.272∗∗∗	-5.272∗∗∗	-2.756	-1.887	-8.502∗∗∗	-8.579∗∗∗
D_DEBT	-4.872∗∗∗	-3.015∗∗	-2.164	-2.376	-1.749	-3.046∗∗	-0.919	-0.811
EA	-6.3142∗∗∗	-6.3199∗∗∗	-13.3465∗∗∗	-13.2558∗∗∗	-6.44∗∗∗	-6.4502∗∗∗	-17.341∗∗∗	-15.213∗∗∗

*Note:* (∗) Significant at the 10%; (∗∗) Significant at the 5%; and (∗∗∗) Significant at the 1%.

**Table 3:** Lag length selection.

Lag	LogL	LR	FPE	AIC	SIC	HQ
0	-533.133	NA	0.197	15.404	15.597	15.480
1	-241.403	525.114	0.0001∗	8.097∗	9.446∗	8.633∗
2	-209.704	51.624∗	0.0002	8.220	10.726	9.215

∗ indicates lag order selected by the criterion.

### 4.1 Cointegration test results

The results of the bound test for cointegration (for the four different models) are reported in Table 4. The bounds test indicates that cointegration exists when BSP, CAR, and ROA are the dependent variables. Thus, the F-statistic of the three models is greater than the upper limit of the corresponding 5% significance level. This shows that the null hypothesis of the bounds test for the three models can be rejected, meaning that there is a long-term relationship for each model. However, the F-statistics in the case where PSC is used as the dependent variable falls between the critical value, which implies that the existence of cointegration is inconclusive.

**Table 4:** ARDL bounds test analysis.

Test statistics				
	Model 1 BSP	Model 2 PSC	Model 3 CAR	Model 4 ROA
F-statistics	4.738∗∗	2.275	4.145∗∗	4.358∗∗

Critical values	10%(0)	10%(1)	5%(0)	5%(1)	1%(0)	1%(1)
	1.99	2.94	2.27	3,28	2.88	3.99

*Notes:* The null hypothesis is no cointegration, ∗, ∗∗, and ∗∗∗ imply 10%, 5%, and 1% respectively.

### 4.2 Long-run relationship

The estimates presented in Table 5 show that in the long run, mobile money transactions is statistically significant and negatively affect banking sector performance (measured as BSP and ROA). The coefficients indicate that a unit increase in mobile money transactions leads to about 1.12 and 0.27 units decrease in BSP and ROA respectively. The relationship is statistically insignificant when banking sector performance is measured using PSC and CAR. The negative relationship derived is in line with the findings of Iheanachor and Ozegbe (2020) and contradicts with Muisyo et al., 2014b, Muisyo et al., 2014a. The returns on assets are a ratio of profitability measuring how much a company’s assets generate profit for it. This outcome supports the claim that a large number of mobile money transactions may lead to most monies being transferred outside of the banking sector for an extended period of time. Thus, people will now use the electronic wallet and see it as a substitute for the traditional bank account. This, therefore, reduces the assets of banks in a form of credits hence a fall in profits that can be created out from the assets. Indeed, the performance of the banking sector can be reduced through a fall in revenues. Thus, most of the urban population shifted from the traditional banking sector towards mobile money services. This action is taken because of both the direct and indirect benefits that mobile money services bring to society. The adoption of mobile money potentially reduces liquidity status of commercial banks hence constraining their key role as a lender to customers. An empirical study in Uganda supports this position as it was revealed that about 36.7% of the variations in the liquidity of Ugandan commercial banks are single-handedly caused by mobile money services (Kamukama and Tumwine, 2012). It was thus, followed that, commercial banks were not able to meet their 20% threshold representing the ratio of total liquid assets to total deposit liability. The case of Uganda appears similar to the Ghanaian case as revealed by the current study. It is, therefore appropriate to conclude that customers are comfortable depositing their funds in the mobile money wallets rather than depositing with the commercial banks.

**Table 5:** The estimated long-run coefficients using the ARDL approach.

Dependent variable	BSP	PSC	CAR	ROA
Regressor	Model 1	Model 2	Model 3	Model 4
MOMO	-1.119∗∗∗ (0.340)	-3.046 (1.89)	0.009 (0.031)	-0.268∗∗ (0.091)
IR	0.426∗∗∗ (0.141)	0.387 (0.659	-0.065∗∗∗ (1.013)	-0.043 (0.036)
SIZE	0.244∗ (0.145)	-0.091 (0.528)	0.020 (0.014)	-0.036 (0.036)
INF	-0.972 (0.646)	-7.608∗∗ (3.008)	0.059 (0.058)	0.309 (0.154)
DDEBT	0.174∗∗∗ (0.058)	0.193 (0.328)	-0.014∗∗∗ (0.005)	0.005 (0.015)
EA	-0.026 (0.167)	0.756 (1.234)	0.021 (0.015)	0.026 (0.046)
C	3.873 (6.760)	42.933 (33.297)	4.170∗∗∗ (6.774)	4.992∗∗∗ (1.752)

*Notes:* ∗∗∗, (∗∗) and (∗) represent significance at 1%, 5% and 10% levels of statistical respectively. Standard errors are in parenthesis.

Accounting for the effect of the controls, interest rate, banking sector size, and domestic debt become positively related to banking sector performance. The coefficient indicates that all other variables held constant, a unit increase in interest rate, banking sector size, and domestic debt results in about 0.43, 0.24, and 0.17 units increase in banking sector performance respectively and these are at least 10 % level of significance in the long run.

It is indicated in Model 2 that inflation significantly reduces banking sector performance measured as the depth. As prices of goods and services increase, the value of money reduces, all other things being equal. Economic theory explains that lenders are disadvantaged as compared to borrowers when the value of money erodes. This explains the fall in credit to the private sector when inflation rises. As argued by Odame-Gyenti and Amaning (2020) the trend of inflation in Ghana can have a detrimental on the credit available to the private sector. Thus, one crucial transmission channel in the Ghanaian context could be through decreased savings and deteriorating deposit mobilisation. Over time, rising inflation decrease real savings hence discouraging loanable funds mobilisation. With individuals, purchasing power is eroded while consumption and the ability to save falls.

Among the controls, interest rate and domestic debt had a negative and significant relationship with banking sector stability. It was revealed that a unit increase in the interest rate and domestic debt leads to 0.065 and 0.014 falls in the banking sector stability in the long run. Increases in interest rate improve the earnings of banks. The banks are able to earn more by benefiting from the difference between the interest paid by banks to customers and the interest earned by the banks by investing. Thus, a bank may make payments to its customers far below the amount it earns from investing in short term interest rates. For the size, the customer base of banks has a high possibility of increasing when the bank in question increases in size. It is argued that individuals perceive it is safer to do business with a bank that has a relatively bigger size (Kulu and Appiah-Kubi, 2021). Funds that commercial banks lend to the public appears as assets to the banks. When repayments are made (especially with interests) at the stipulated time, the commercials banks are able to generate some amount of money from the loan made. Indeed, liquidity and asset ratio are improved in the process. Empirically, Janda and Kravtsov (2017) concludes that growth in public debt within the Central and Eastern European countries positively influence the performance and efficiency of the banking sector.

### 4.3 Short-run relationship

Table 6 shows that in the short run, mobile money transactions hurt banking sector performance. This relationship is statistically significant at 1 %. However, in Model 3, it is evident that banking sector performance measured by stability (CAR) is positively influenced by mobile money transactions. Tiriongo and Wamalwa (2020) provide a contradicting argument as they found a fall in the stability of the Kenyan banking system. Again, as argued by Kipkemboi and Bahia (2019), a positive relationship is possible when mobile money accounts are linked to the formal bank accounts leading to a fall in the unit cost of the financial services which makes it possible for banks to expand their customer base and products offered. In the short-run, the effect of mobile money transactions on the depth and efficiency are statistically insignificant.

**Table 6:** The estimated short-run coefficients using the ARDL approach.

Dependent variable	BSP	PSC	CAR	ROA
Regressor	Model 1	Model 2	Model 3	Model 4
D(MOMO)	-2.507∗∗∗ (0.850)	-0.321 (0.431)	0.130∗∗ (0.062)	-0.428 (0.260)
D(IR)	0.276∗ (0.161)	0.110 (0.080)	-0.027∗∗ (0.012)	-0.009 (0.048)
D(SIZE)	0.102 (0.062)	0.009 (0.031)	-0.008∗ (0.005)	0.012 (0.019)
D(INF)	-2.076∗∗∗ (0.624)	-8.262∗∗∗ (0.314)	0.119∗∗ (0.046)	0.197 (0.192)
D(DDEBT)	0.125∗∗ (0.060)	0.112∗∗∗ (0.030)	-0.013∗∗∗ (0.004)	-0.007 (0.018)
D(EA)	-0.065 (0.095)	-0.082 (0.050)	0.020∗∗∗ (0.007)	0.019 (0.029)
ECM (-1)	-0.763∗∗∗ (0.114)	-0.076∗∗∗ (0.020)	-0.675∗∗∗ (0.099)	-0.847∗∗∗ (0.138)
$\bar{R}2$	0.750	98.83	0.776	0.461
F-Stat	19.524∗∗∗	323.029∗∗∗	20.599∗∗∗	7.641∗∗∗
DW-Stat	2.341	2.055	1.772	2.193
Diagnostic Tests				
Serial Correlation	1.607 [0.211]	0.128 [0.880]	0.303 [0.740]	0.709 [0.497]
Heteroskedasticity	1.708 [0.104]	3.873 [0.001]	0.992 [0.466]	1.144 [0.350]
Normality	2.671 [0.263]	17.140 [0.0002]	16.395 [0.002]	294.695 [0.000]
Functional Form	0.081 [0.778]	135.443 [0.000]	0.480 [0.492]	0.258 [0.614]

*Notes:* ∗∗∗, ∗∗, and ∗ represent 1%, 5%, and 10% level of significance respectively (…) are the standard errors, and […] are prob. values.

Just as found in the long-run estimates, it is also found that inflation and domestic debt are statistically significant and respectively have a negative and a positive effect on banking sector performance in the short run. Again, the study also found that banking sector size and interest rate are statistically significant and negatively affect banking sector efficiency.

The estimated ECT−1 coefficients are negative and statistically significant at 1% for all specifications (see Table 6). The negative and significant coefficients of the ECT−1 confirm the cointegration results. The result shows a high speed of adjustment of convergence to the long-run equilibrium every year after a short-run shock. Specifically, equilibrium, in the long run will adjust by approximately 76 percent, 7.6 percent, 67 percent, and 84 percent in Models 1, 2, 3, and 4, respectively, every year after any shock observed in the short run.

The diagnostic tests are presented in Table 6. In order to test the robustness of the models, we conduct diagnostic and stability tests. In this vein, we test for the presence or otherwise absence of misspecified functional form, normality, heteroskedasticity, and autocorrelation in the ARDL model specification. Both specifications of banking sector performance equations are found to be stable over the sample period. This is confirmed by the plots of the CUSUM and CUSUMSQ tests as proposed by Brown et al. (1975) and suggested by Pesaran et al. (2001) within the ARDL framework. In both specifications, the CUSUM and CUSUMSQ residuals lines lie within the 5 percent critical value bounds (see Appendix D), implying stability in the models. Hence, it can be concluded that the regression equations are stable throughout the sample period considered.

### 4.4 Impulse responses results

**Figure 1:**
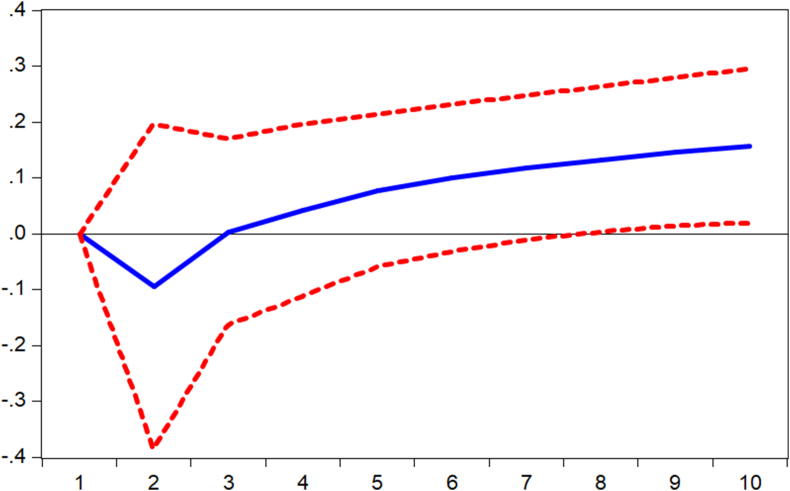
Response of CAR to MOMO Innovation using Cholesky (d.f. adjusted) Factors.

**Figure 2:**
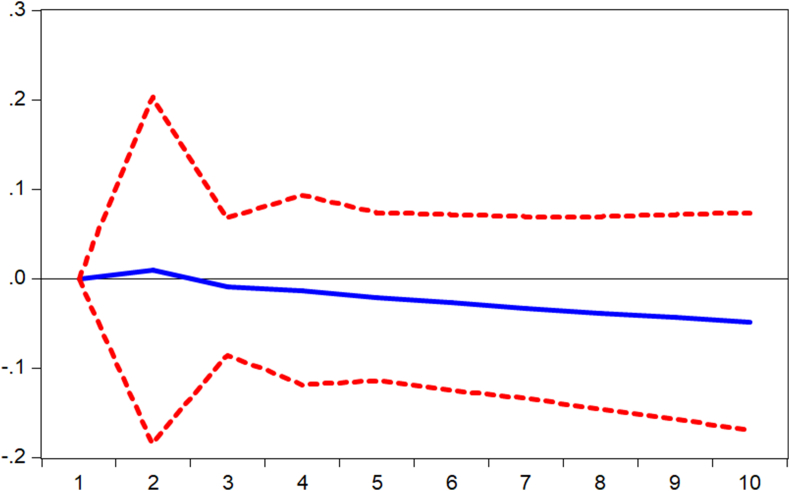
Response of BSP to MOMO Innovation using Cholesky (d.f. adjusted) Factors.

**Figure 3:**
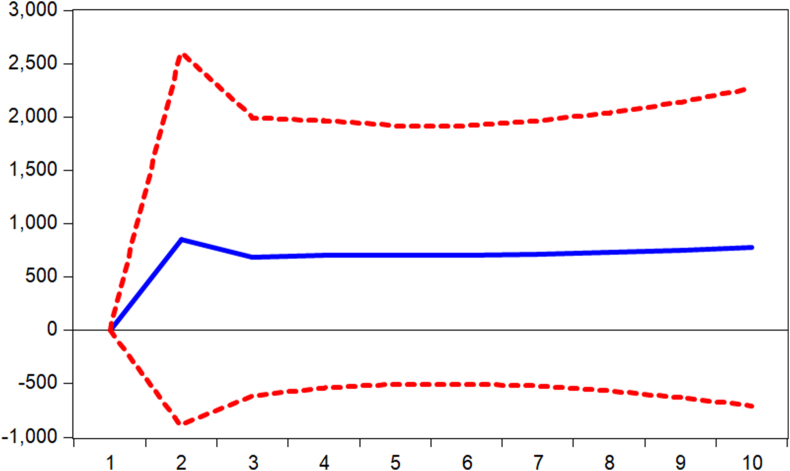
Response of ROA to MOMO Innovation using Cholesky (d.f. adjusted) Factors.

**Figure 4:**
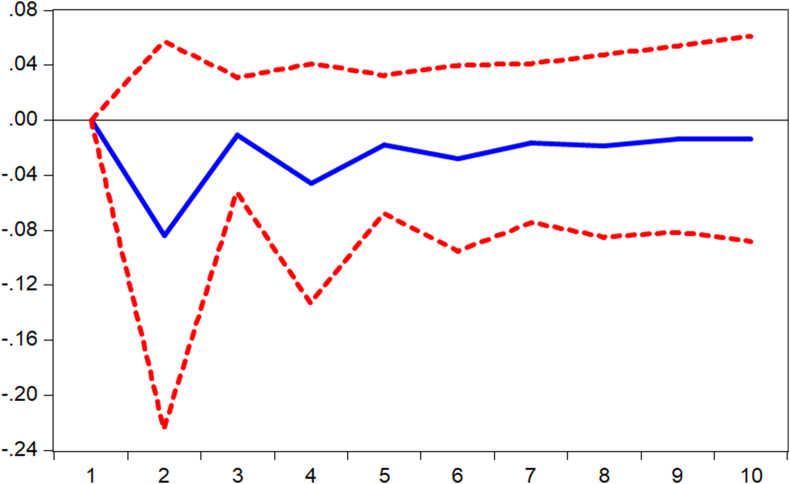
Response of PSC to MOMO Innovation using Cholesky (d.f. adjusted) Factors.

Impulse responses are performed to complement the findings from the regression models. Thus, we verify how shocks in mobile money transactions (MOMO) are responded to by the various measures of banking sector performance. Figure 1 displays the response of banking sector performance measured by capital adequacy ratio (stability) to shocks in mobile money transactions. It is observed that the performance responds negatively to shocks from the first to the third period and begins to respond positively afterward. Again, it is observed from Figures 2 and 3 that the banking sector efficiency (ROA) and the composite index of the banking sector performance respond negatively to shocks in mobile money transactions. Thus, Figure 3 shows that shocks in mobile money transactions erode the returns on assets of commercial banks. However, as evident in Figure 4, with an impulse in the mobile money transaction, depth of the banking sector responds positively. This confirms the argument by Ahmed and Wamugo (2018) that mobile money complements internet banking hence an increase in credit available to the private sector.

## 5 Conclusions and policy recommendations

This study examined the effect of mobile money transactions on banking sector performance in Ghana. Prior studies have documented the role of systemic risk and bank-specific shocks for financial system stability with little focus on the role of mobile money transactions on banking sector performance. We examine this relationship using a composite index measuring banking sector performance as well as the disaggregated components – capital adequacy ratio, banks credit to the private sector and return on assets. Our results show that mobile money transaction has a detrimental effect on the composite index of banking sector performance, while we found mixed results for some of the respective components. Again, we found that interest rate, domestic debt exerts a significant positive effect on banking sector performance in both the short and long-run. Based on the findings, it is concluded that mobile money transactions affect the performance of the banking sector. However, the implication differs from one measure to the other in terms of direction and magnitude of influence. It is therefore recommended that commercial banks should have a partnership with the mobile money operators and settle on a number of services that will enable customers to simultaneously use both the mobile money and banking services also, there is the need for banks to exercise much caution in their decision to consider adopting mobile money-based services when designing their business models.

## Declarations

### Author contribution statement

Evans Kulu; Emmanuel Gbolonyo: Conceived and designed the experiments; Analyzed and interpreted the data; Wrote the paper.

Alexander Opoku: Conceived and designed the experiments; Performed the experiments; Analyzed and interpreted the data; Contributed reagents, materials, analysis tools or data; Wrote the paper.

Mark Anthony Tayi Kodwo: Performed the experiments; Contributed reagents, materials, analysis tools or data; Wrote the paper.

### Funding statement

This research did not receive any specific grant from funding agencies in the public, commercial, or not-for-profit sectors.

### Data availability statement

Data will be made available on request.

### Declaration of interest’s statement

The authors declare no conflict of interest.

### Additional information

No additional information is available for this paper.

## Figures and Tables

**Table:** 

Variable	Symbol	Measurement
Banking sector performance index	BSP	Composite index
Banking sector depth	PSC	Private Sector Credit to GDP
Efficiency	ROA	Return On Assets
Stability	CAR	Capital adequacy ratios
Mobile Money Transactions	MOMO	Amount of mobile money transactions in million Ghana cedis
Interest rate	IR	Monetary policy rate
Size of Banking sector	SIZE	Core liquid assets to total assets (%)
Inflation rate	INF	Consumer price index
Domestic debt	DDEBT	Gross domestic debt as a percentage of GDP
Economic activity	EA	Composite Index of Economic Activity (Real Growth) (%)

**Table:** 

				Number of obs = 72
				Number of comp. = 3
				Trace = 3
			Rotation: (unrotated = principal)	Rho = 1.0000
Component	Eigenvalue	Difference	Proportion	Cumulative
Comp1	1.314	0.130	0.438	0.438
Comp2	1.184	0.682	0.395	0.833
Comp3	0.502	.	0.167	1.000

**Table:** 

Variable	Comp 1	Comp 2	Comp 3	Unexplained
ROA	-0.733	-0.301	0.610	0
CAR	0.675	-0.433	0.597	0
PSC	0.084	0.850	0.521	0

**Table:** 

Probability	BSP	MOMO	IR	SIZE	INF	DDEBT	EA
BSP	1						
MOMO	-0.586	1					
	0.000						
IR	0.749	-0.912	1				
	0.000	0.000					
SIZE	0.570	-0.826	0.812	1			
	0.000	0.000	0.000				
INF	0.446	-0.600	0.650	0.368	1		
	0.000	0.000	0.000	0.002			
DDEBT	-0.483	0.502	-0.585	-0.476	-0.425	1	
	0.000	0.000	0.000	0.000	0.000		
EA	0.039	0.097	-0.025	-0.034	-0.010	0.200	1
	0.745	0.418	0.833	0.779	0.937	0.092	

**Figure:**
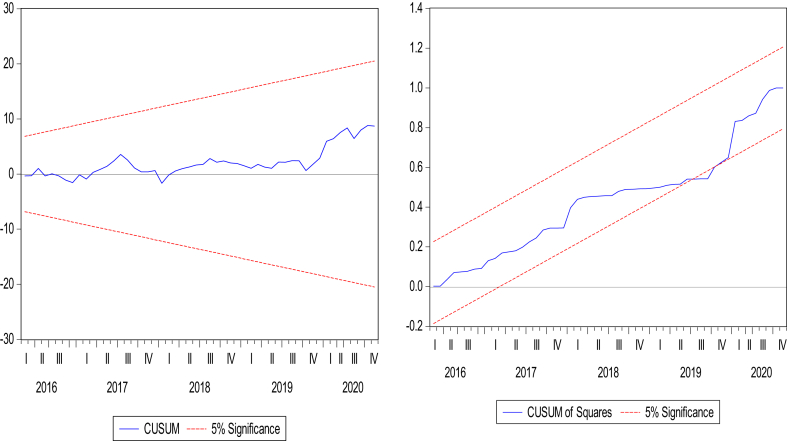


**Figure:**
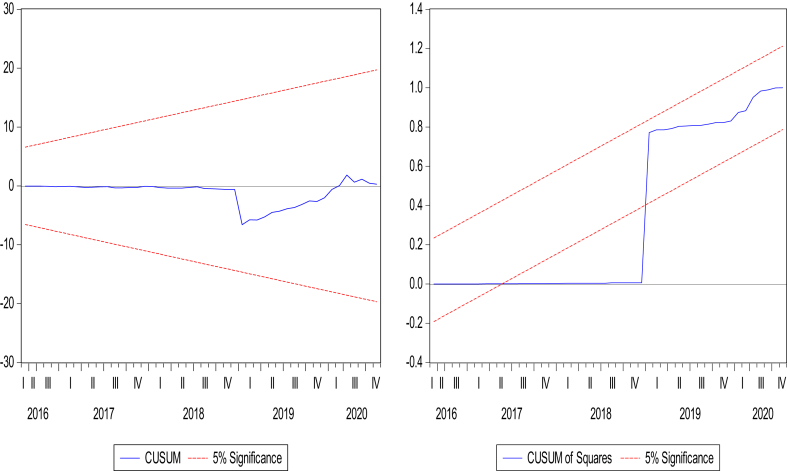


**Figure:**
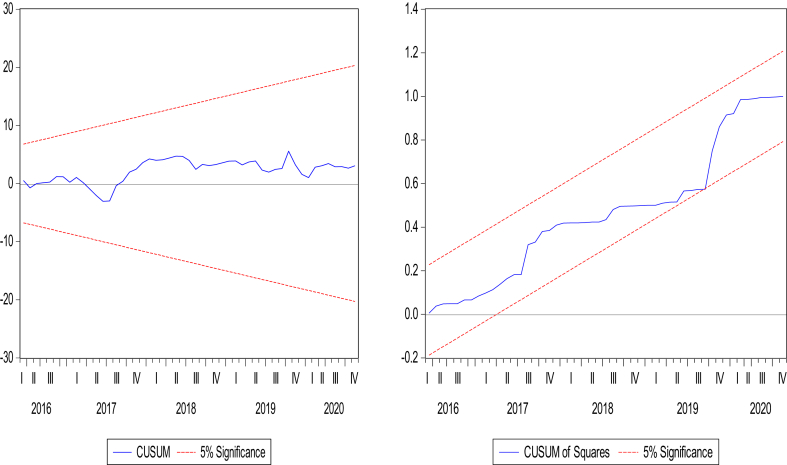


**Figure:**
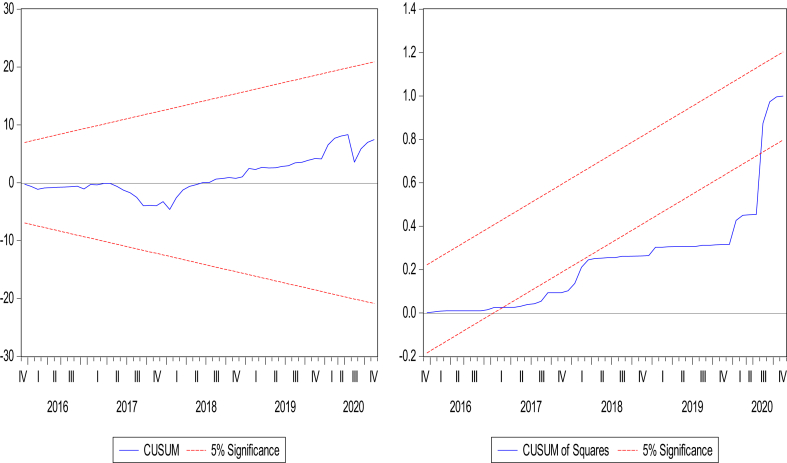


## Appendices

### Appendix A Variable Measurement, Description, and Source

*Source:* Authors' construct (2021).

## Appendix B Principal Components/Correlation

Principal components (eigenvectors).

## Appendix C Correlation matrix

*Source:* Authors' Construct.

## Appendix D CUSUM and CUMSQ for BSP, PSC, ROA, CAR

BSP

PSC

CAR

ROA
